# Habitat and climatic associations of climate‐sensitive species along a southern range boundary

**DOI:** 10.1002/ece3.10083

**Published:** 2023-05-17

**Authors:** Evan C. Wilson, Stella Cousins, Dwayne R. Etter, John M. Humphreys, Gary J. Roloff, Neil H. Carter

**Affiliations:** ^1^ School for Environment and Sustainability University of Michigan Ann Arbor Michigan USA; ^2^ Michigan Department of Natural Resources Lansing Michigan USA; ^3^ Department of Fisheries and Wildlife Michigan State University East Lansing Michigan USA; ^4^ United States Department of Agriculture, Agricultural Research Service Sidney Montana USA

**Keywords:** American marten, Gaussian Random Field, INLA, Lowland Riparian, Moose, Ruffed Grouse, Snowshoe Hare, species distribution model, winter habitat indices, upland spruce‐fir

## Abstract

Climate change and habitat loss are recognized as important drivers of shifts in wildlife species' geographic distributions. While often considered independently, there is considerable overlap between these drivers, and understanding how they contribute to range shifts can predict future species assemblages and inform effective management. Our objective was to evaluate the impacts of habitat, climatic, and anthropogenic effects on the distributions of climate‐sensitive vertebrates along a southern range boundary in Northern Michigan, USA. We combined multiple sources of occurrence data, including harvest and citizen‐science data, then used hierarchical Bayesian spatial models to determine habitat and climatic associations for four climate‐sensitive vertebrate species (American marten [*Martes americana*], snowshoe hare [*Lepus americanus*], ruffed grouse [*Bonasa umbellus*] and moose [*Alces alces*]). We used total basal area of at‐risk forest types to represent habitat, and temperature and winter habitat indices to represent climate. Marten associated with upland spruce‐fir and lowland riparian forest types, hares with lowland conifer and aspen‐birch, grouse with lowland riparian hardwoods, and moose with upland spruce‐fir. Species differed in climatic drivers with hares positively associated with cooler annual temperatures, moose with cooler summer temperatures and grouse with colder winter temperatures. Contrary to expectations, temperature variables outperformed winter habitat indices. Model performance varied greatly among species, as did predicted distributions along the southern edge of the Northwoods region. As multiple species were associated with lowland riparian and upland spruce‐fir habitats, these results provide potential for efficient prioritization of habitat management. Both direct and indirect effects from climate change are likely to impact the distribution of climate‐sensitive species in the future and the use of multiple data types and sources in the modelling of species distributions can result in more accurate predictions resulting in improved management at policy‐relevant scales.

## Graphical abstract

Climate and habitat are important drivers of species' distributions. We used a variety of data sources to fit Bayesian hierarchical models and determine associations between habitat, climatic and anthropogenic conditions for four climate‐sensitive species' in Northern Michigan and predict contemporary distributions. Species were associated with multiple climate‐sensitive habitat classes, while temperature itself was a stronger predictor than more mechanistic variables of snow cover.

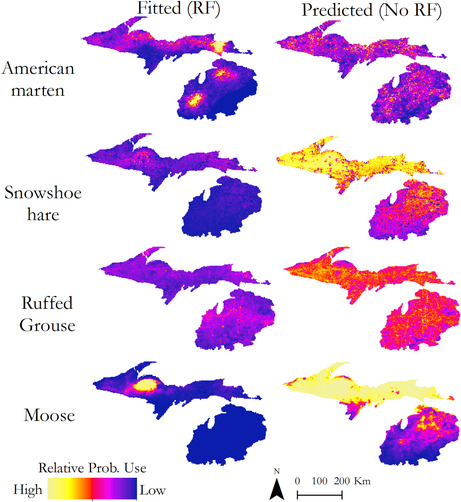

## 1 INTRODUCTION

Climate change is altering species distributions and shifting geographic ranges for many species along elevational and latitudinal gradients (Parmesan et al., 1999; Williams & Blois, 2018). These range shifts can result in altered community assemblages and novel, no analogue communities (Radeloff et al., 2015), particularly along range boundaries, as climate‐sensitive species decline along trailing edges and other species expand ranges along leading edges (Williams & Jackson, 2007). Historically, research on the drivers of species range extents has focused on habitat as the dominant driver of species range extents; however, recent studies have indicated that particularly in climate‐sensitive species and regions along southern range boundaries, climate may be equally as important and growing in importance as a driver of species range limits (Reich et al., 2022; Sultaire et al., 2016). These two drivers—habitat and climate change—do not operate independently and there is considerable overlap between them at both macro‐ and microscales. Climate change can alter hydrological patterns, fire regimes, and other mechanisms that directly affect biotic and abiotic components of habitat (Halofsky et al., 2020; Trenberth, 1999). Hence, species may experience direct physiological effects from altered precipitation or temperature patterns, but also indirect effects due to climate change impacts on habitats. A better understanding of the interactive effects of these drivers on species distributions will elucidate the mechanisms underlying range shifts and be important for managing biodiversity under global change.

Ecological communities and wildlife populations along ecosystem boundaries are often among the first to experience impacts of climate change (Gilman et al., 2010). The Northwoods region, spanning the Upper Great Lakes region of North America, contains one such ecological boundary that delineates where southern deciduous forests and northern coniferous (“boreal”) forests meet (Andersen, 2005). While the boreal forest is one of the world's largest biomes (Gauthier et al., 2015), dynamics along the southern boundary can often differ greatly from those in the core biome, and the effects of climate change are already having a pronounced effect on community dynamics (Wilson et al., 2022). For example, subnivium habitat—the zone between fallen snow and terrain (Pauli et al., 2013)—is becoming more unstable along southern range boundaries due to the effects of climate change (Thompson et al., 2018). Because many species make use of the subnivium during winter months, and rely on the relatively stable temperatures within to persist during winter (Zuckerberg & Pauli, 2018), the interaction of changing snow conditions and temperatures can negatively impact snow‐adapted species. Indeed, recognition of the role of snow as habitat has prompted the creation of spatially and temporally explicit winter habitat indices to characterize dynamic snow conditions in space and time (Gudex‐Cross et al., 2021).

In addition to climate‐sensitive habitats, the Northwoods region is also home to multiple climate‐sensitive wildlife species (Hoving et al., 2013), including important game species such as American marten (*Martes americana*), moose (*Alces alces*), snowshoe hare (*Lepus americanus*), and ruffed grouse (*Bonasa umbellus*). These species provide numerous cultural and ecosystem services yet are distinctly vulnerable to climate change. Martens rely on deep snow to avoid competition and direct predation by larger mesocarnivores (e.g., bobcat [*Lynx rufus*], coyote [*Canis latrans*], fisher [*Pekania pennanti*]) that have heavier foot loading and more difficulty moving through snow (Crête & Larivière, 2003). Additionally, loss of preferred prey species such as red‐backed voles (*Myodes gapperi*) linked to habitat and climate change can negatively affect marten populations (Carlson et al., 2014; Scott et al., 2022). Moose experience direct effects from warming temperatures through increased heat stress, increased levels of parasitism by ecto‐ and endoparasites, and increased competition with white‐tailed deer (*Odocoileus virginianus*), which are historically limited by deep snows (Weiskopf et al., 2019). Additionally, moose may experience indirect effects due to negative impacts of climate change on boreal forest types in which they reside and forage (Weiskopf et al., 2019). Snowshoe hares have experienced northward range contractions along trailing edge boundaries (Burt et al., 2017; Diefenbach et al., 2016; Sultaire et al., 2016), partially driven by increased predation rates linked to mismatch in the timing of coat color molts with attenuated snow cover due to climate change (Wilson et al., 2019; Zimova et al., 2016). Ruffed grouse use the subnivium, the below‐snow refugium that maintains a stable thermal environment during winter months, as a method to avoid predators and conserve energy during winter months (Pauli et al., 2013). The loss of consistent deep snow along southern range boundaries results in increased stress in individuals and declines in survival (Shipley et al., 2020). Despite extensive geographic overlap in distributions and general associations with colder temperatures and northern forest habitats, specific habitat requirements and mechanisms linking climate to vital rates can differ greatly among species, resulting in challenges for simultaneous management of these species in the region. For example, while early successional forests are preferred habitat for ruffed grouse (Rusch et al., 2020) and can increase survival in snowshoe hares (Wilson et al., 2019), marten prefer mature forest with structural complexity (Chapin et al., 1997). Therefore, there is a need to understand how species differentially respond to climate and habitat and identify areas of overlap where management action can have the broadest benefit for multiple species and ecosystems.

**FIGURE 1:**
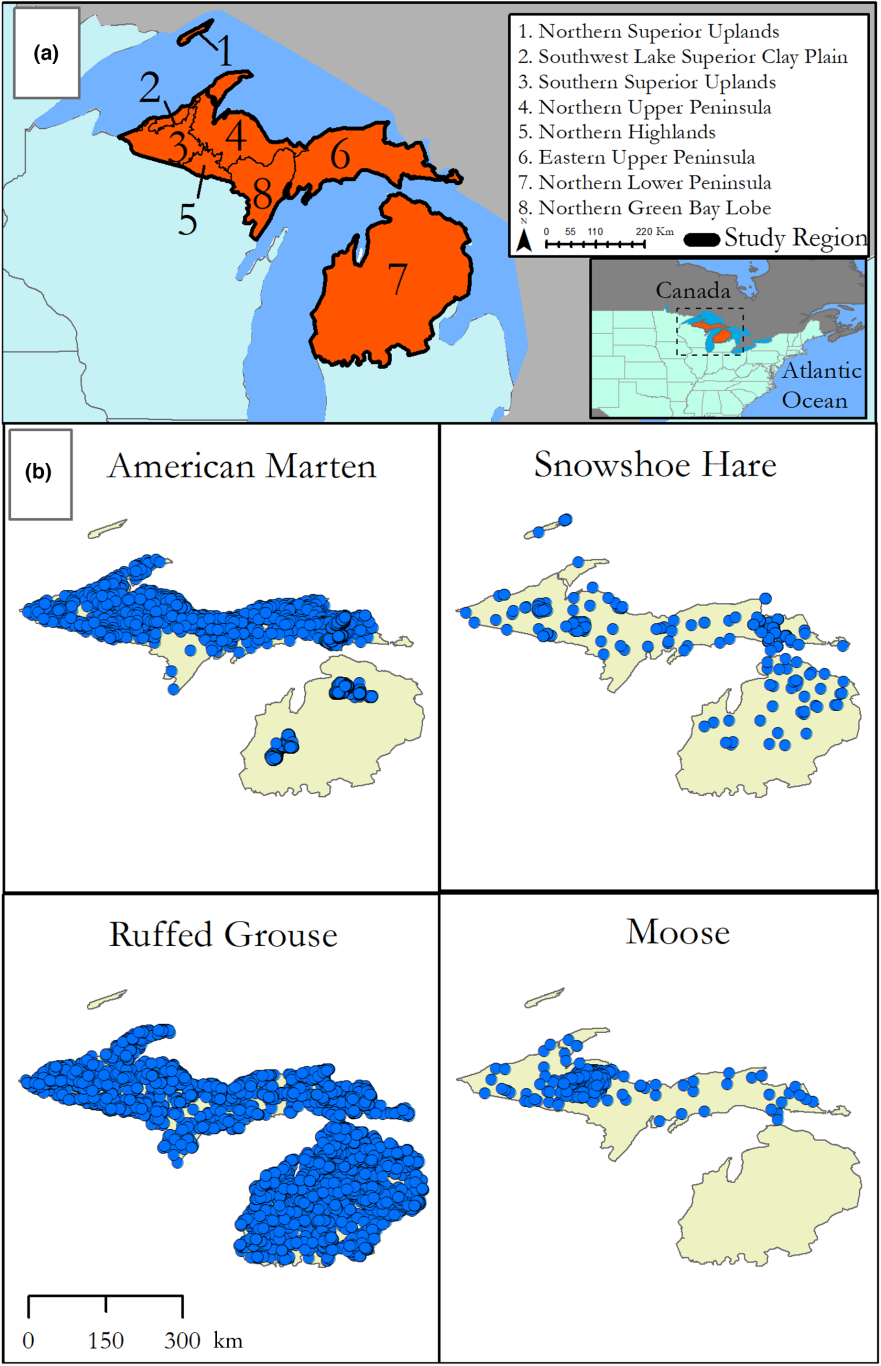
Maps of study areas with occurrence data of focal species. (a) Map of Northwoods region of Michigan, with ecoregions labelled (per Albert, 1995), the greater Northwoods area is outlined in orange. (b) Maps of occurrence data collected and used to construct distribution models for Ruffed Grouse (*Bonasa umbellus*), moose (*Alces alces*), American marten (*Martes americana*), and snowshoe hare (*Lepus americanus*).

Here, we modeled the effects of climate, climate‐sensitive forest cover types, and direct anthropogenic effects on the abovementioned four iconic vertebrate species in Michigan—a geographically unique state split by the Great Lakes into upper and lower peninsulas (Figure 1a). Our models utilized the Integrated Nested Laplacian Approximation (INLA; Rue et al., 2009) with Stochastic Partially Differential Equation (Lindgren et al., 2011), which is a computationally efficient and accurate method for analyzing spatial data and accounting for the effects of spatial autocorrelation. For our models, we integrated multiple forms of animal occurrence data, including radio telemetry, harvest records, and observations recorded in conjunction with citizen science programs. While data collected through formal field survey has inherent advantages, namely standardized sampling methods allow for more accurate modeling of detection probabilities, it is often expensive and yields a relatively small amount of data compared with community science data, which lack repeat sampling but can yield large amounts of data rather cheaply. Focusing solely on one form of data can often yield misleading results (Kamp et al., 2016), whereas incorporating both can yield more robust estimates. To capture changing dynamics of snow as habitat, we also incorporated recently developed winter habitat indices, which are intended to more accurately describe the mechanisms affecting individual fitness (Gudex‐Cross et al., 2021). Finally, we spatially predicted the distributions of our four focal species given model results in order to visualize the geographic heterogeneity of habitat suitability.

We predicted that multiple species would be associated with the same forest cover types, potentially allowing for mutually beneficial management of forest habitat. We predicted that we would observe species‐specific relationships with climate variables, but that measures of snow conditions (i.e., winter habitat indices) would outperform more general measures of climate, such as temperature, particularly for marten, grouse, and hares—species with recognized interactions with snow. Finally, we predicted that the southern extent and contiguity of distribution would vary between species as species respond to different habitat and climatic factors. These distributional models provide insight into effects of climate, habitat, and direct anthropogenic activity on climate‐sensitive species distributions and allow for future projections of the direct and indirect effects of climate change on climate‐vulnerable species in this region. Knowledge of how climate affects wildlife species directly and indirectly, via habitat alteration, can facilitate efficient use of resources to manage habitat at policy‐relevant scales (e.g., local and state level) and buffer species from the negative impacts of climate change resulting in observed range shifts.

## 2 METHODS

### 2.1 Study area

Our study area comprised the Northern Lower Peninsula and Upper Peninsula of Michigan (Ecological section VII per Albert, 1995; Figure 1a), which combined is considered part of the Northwoods ecosystem. While these two peninsulas are unconnected and connectivity between populations in the lower and upper peninsulas is unlikely, they do share similar regulatory and conservation history. This region is highly seasonal with mean annual temperatures of 6.2°C, while mean summer temperatures and mean winter temperatures were 18.3°C and −6.4°C, respectively. Annual precipitation ranges from 71 to 86 cm across the study area, with lake effect snow being a defining feature across much of the coastal region and annual average snowfall ranging from 101 to 356 cm. Elevation changes in the region were minimal with a mean elevation of 296 m (SD = 88.6) and minimum and maximum elevations of 173 m in the southeast and 613 m in the western upper peninsula, respectively.

### 2.2 Wildlife occurrence data

We obtained species occurrence data from a variety of sources (Figure 1b). Occurrence data reflected only presence of a species, and made no assumptions about population density. For marten from the Upper Peninsula, we used harvest data from trappers collected by the Michigan Department of Natural Resources (MDNR) between 2000 and 2020. As part of license requirements, successful trappers are required to report the harvest location within a 1 square mile (2.6 km^2^) quadrant. We supplemented these records with locations of resting sites and telemetry locations from research studies by the Little River Band of the Ottawa Indians (Sanders et al., 2017) and Sault Ste. Marie Tribe of Chippewa Indians (Roloff et al., 2020), and citizen science observations obtained from GBIF (https://doi.org/10.15468/dl.6zbg5c) and eMammal (Cove et al., 2021). For ruffed grouse we used eBird checklists, detailing both detections and nondetections, collected between January 1, 2000, and December 31, 2020 (eBird Basic Dataset, 2021). For moose from the Upper Peninsula, we used observations from aerial flights, observations, and vehicle collisions collected by MDNR, supplemented by community science observations from GBIF (https://doi.org/10.15468/dl.yndr72). For snowshoe hare, we combined observations from track and trail camera surveys compiled from multiple sources (Burt et al., 2016; Sultaire et al., 2022), and community science observations from eMammal (Cove et al., 2021) and GBIF (https://doi.org/10.15468/dl.yndr72).

### 2.3 Predictors

Seven forest cover types were considered to be under moderate‐to‐high vulnerability from climate change (Handler et al., 2014). To represent them, we used a spatial layer containing the sum of the basal area (m^2^/ha) for two to five tree species representative of each cover type (Table 1; Dickmann & Leefers, 2003; Handler et al., 2014). Basal area is sum of cross‐sectional surface areas of each live tree within a plot measured at breast height (Bettinger et al., 2017). Basal area for tree species were obtained from a US Forest Service data product containing raster maps of live tree basal area for tree species at a 250‐m resolution (Wilson et al., 2013), obtained by integrating vegetation phenology from MODIS imagery and field plot data from the Forest Inventory and Analysis database between 2000 and 2009 (Wilson et al., 2013). This dataset was validated for spatial accuracy by comparing modelled data to observed data from Forest Inventory and Analysis (FIA) plots and comparing accuracy metrics (Riemann & Wilson, 2014).We created separate layers for each forest cover type by summing the basal areas for all representative species within each cell (Figure S1). Basal area is a common measure of tree density used in forestry to represent aboveground biomass (Bettinger et al., 2017) as it contains information on number of trees and size (Balderas Torres & Lovett, 2013), and is often used to represent forest structure in resource selection models (Irwin et al., 2020; Parsons et al., 2019).

**TABLE 1:** Climate‐sensitive forest types of northern Michigan used as predictors in the individual species distribution models, together with their component tree species and vulnerability ratings (per Handler et al., 2014).

Forest type	Tree species		Vulnerability (per Handler et al., 2014)
	Common name	Scientific name	
Upland spruce‐fir	Balsam fir	*Abies balsamea*	High
	White spruce	*Picea glauca*	
	White pine	*Pinus strobus*	
Jack pine‐red pine	Jack pine	*Pinus banksiana*	High‐moderate
	Red pine	*Pinus resinosa*	
White pine‐red pine	White pine	*Pinus strobus*	High‐moderate
	Red pine	*Pinus resinosa*	
Lowland conifers	Black spruce	*Picea mariana*	High‐moderate
	Eastern hemlock	*Tsuga canadensis*	
	Northern white cedar	*Thuja occidentalis*	
	Tamarack	*Larix laricina*	
Aspen‐birch	Quaking aspen	*Populus tremuloides*	Moderate
	Big‐toothed aspen	*Populus gradidentata*	
	Balsam poplar	*Populus balsamifera*	
	Paper birch	*Betula papyrifera*	
Northern hardwoods	American basswood	*Tilia americana*	Moderate
	Sugar maple	*Acer saccharum*	
	Northern red oak	*Quercus rubra*	
	Red maple	*Acer rubrum*	
	American beech	*Fagus gradifolia*	
Lowland riparian hardwoods	Silver maple	*Acer saccharinum*	Moderate
	Green ash	*Fraxinus pennsylvanica*	
	Black ash	*Fraxinus nigra*	
	Red maple	*Acer rubrum*	
	Northern white cedar	*Thuja occidentalis*	

We used two types of climate predictors to model species occurrence. First, we used three different metrics of temperature: mean annual temperature, mean temperature of the warmest quarter (i.e., summer), and mean temperature of the coldest quarter (i.e., winter). Mean values for temperature metrics from 1970 to 2000 were obtained at a 30‐s resolution from WorldClim (Fick & Hijmans, 2017). Next, we used three winter habitat indices developed to represent more functional aspects of winter ecology, specifically snow season length, snow variability, and duration of frozen ground without snow cover (Gudex‐Cross et al., 2021). Winter habitat indices were developed from MODIS satellite data and validated by comparing modelled output to that of NOAA weather stations (Gudex‐Cross et al., 2021). Snow season length was represented in days as the difference between the adjusted Julian date (DOY 1 = 1 August) of last snow detection and adjusted Julian date of first snow detection (Gudex‐Cross et al., 2021). Frozen ground without snow was represented as the percentage of the total frozen days (minimum temperature < −4°C) where snow was absent (Gudex‐Cross et al., 2021). Snow cover variability was represented by the percentage of observations in which snow cover status had changed (i.e., present/absent) from the previous observation (Gudex‐Cross et al., 2021). Mean values of winter habitat indices from 2003 to 2020 were obtained at 500‐m resolution (Figure S2).

We used four different metrics to represent direct effects of human activity and urbanization: housing density, road density, distance to major roads, and distance to conservation lands. Both housing and road density are well‐established indices of human density (Forman et al., 2003; Lewis et al., 2011), and represented levels of human activity and development within the study area. Highways and other major roads can also serve as a barrier to dispersal and animal movements (Forman et al., 2003). Additionally, citizen science and other unstructured data sources frequently have detection biases in relation to roads due to increased accessibility (Cretois et al., 2021). Conservation and recreation lands comprised both private and public lands managed for either conservation (e.g., state wildlife areas and national forests) or recreation (e.g., hunting clubs and golf courses). Both protected areas and working lands (i.e., rangeland, agriculture, and forested areas used in commercial enterprises) can provide suitable habitat for a variety of species within human dominated landscapes and may act as refugia for climate‐sensitive species (Pacifici et al., 2020; but see Parks et al., 2023). Housing density was represented by the 2010 household density (households/km^2^) value obtained from a dataset detailing census block‐level housing change between 1990 and 2010 (Martinuzzi et al., 2015). Road density (m/km^2^) was represented by the cumulative length of road features obtained from Michigan's Open GIS Data portal (Allroads v17a; Michigan DOT, 2015) in each 1‐km resolution grid cell. Distance to major roads (m) was represented by the distance to nearest road with a National Functional Classification value between 1 and 4 (Interstates, other freeways, other principal arterials, and minor arterials) (Allroads (v17a); Michigan DOT, 2015). Distance to conservation lands (m) was represented by the distance to the nearest conservation or recreation lands under all jurisdictions (Ducks Unlimited, 2021).

### 2.4 Analysis

We implemented a Bayesian hierarchical approach in INLA, using the R package *INLA* (Rue et al., 2009) to model occurrence of each focal species using a generalized linear mixed model. INLA uses the Stochastic Partial Differential Equations (SPDE) approach for the spatial effect, approximating a Gaussian Random Field where the correlation between locations is Matèrn (i.e., covariance between two points is related to the distance between points). The random fields served as error terms to measure spatial autocorrelation and other uncertainty not explained by fixed effects included in the models. We constructed a random field (RF) by creating a two‐dimensional triangulated mesh using guidelines provided by Lindgren and Rue (2015). We used mesh vertices to represent background locations, and supplemented these points with a regularly spaced grid over terrestrial regions.

We extracted values from each predictor layer for occurrence points and all background points. All continuous predictors were scaled by subtracting the mean and dividing by standard deviation. The dataset for each species was then divided randomly into training (80%) and testing (20%) subsets. Observations of our focal species are modelled as a Bernoulli point process such that Z(s) indicates the presence (1) or absence (0) of the species at location s, with the probability of presence given as $\pi_{s}$. This relationship can be expressed as,

(1) $$Z\left(s\right)\sim \text{Bernoulli}\;\left(\pi_{s}\right)$$

$$\text{logit}\left(\pi_{s}\right)=\beta_{0}+\Sigma \left(X_{s}\beta_{\text{habitat}}\right)+X_{s}\beta_{\text{climate}}+\Sigma \left(X_{s}\beta_{\text{anthropogenic}}\right)+{\mathrm{RF}}_{s}+\in_{\mathrm{NN}\left(s\right)}$$

where $\beta_{0}$ is the intercept term, $X_{s}$ is a vector of values for each predictor, $\beta_{\text{habitat}}$, $\beta_{\text{climate}}$, and $\beta_{\text{anthropogenic}}$ are the coefficients for each habitat, climate, and anthropogenic predictors respectively, ${\mathrm{RF}}_{s}$ is the spatially structured random effect and $\in_{\mathrm{NN}\left(s\right)}$ is a predictor included to capture spatially structured effects of aggregated individuals (i.e., clustering) (Humphreys et al., 2017).

We used a multistage process for model selection. First, we checked for issues with collinearity using the R package *corrplot*, and excluded any potential models that contained predictors with >0.6 Pearson's correlation coefficient values. Subsequently, the first stage of model selection involved evaluating all possible combinations of habitat predictors. We then took the top habitat model, added climate predictors univariately and selected the top climate and habitat model. Finally, we added all combinations of anthropogenic predictors to the top climate and habitat model. Models were ranked using Watanabe–Akaike information criterion (WAIC) at each stage, and while models with equivalent support (≤2 WAIC) were noted only the top‐ranked model was transferred to the next stage of model selection. Watanabe–Akaike information criterion is a fully Bayesian information criterion, and while issues remain regarding the use of WAIC in structured datasets such as spatial models (Gelman et al., 2014), it is frequently used in hierarchical spatial modelling (see Leach et al., 2016; Sultaire et al., 2022; Williamson et al., 2022) and is preferred to other criteria such as DIC (Doser et al., 2022; Duncan & Mengersen, 2020). Coefficients of all top‐ranked models were examined, and statistical significance was determined by comparing 95% credible intervals of the effect coefficients to zero.

To facilitate the interpretation of parameters, we converted beta coefficients for fixed effects to relative selection strength values by applying an exponential function to each coefficient value (Avgar et al., 2017). Relative selection strengths (RSS) can be interpreted as the relative intensity of use of the fixed effect by the species between locations that differ by one unit of the fixed effect if all other effects are equal (Fieberg et al., 2021). To visualize these relationships, we predicted relative probability of presence values at all available locations using the fixed effects and random field values for final top‐ranked model of each species and plotted these values against the variable values for each predictor at the available location (Avgar et al., 2017). Relative probability of presence was scaled from zero to one by dividing each value by the maximum value. We then used the gam function in the R package *mgcv* (Wood & Wood, 2015) to fit a function to the available data, while using smoother parameters <5 to avoid overparameterization (Perrig et al., 2020).

To compare fitted and predicted spatial models (i.e., with and without inclusion of a spatially explicit random field), we created two predictive surfaces of the study area for each species at a 2000‐meter resolution, one model containing all fixed effects and a random spatial effect (RF), and a second model containing just the fixed effects (No RF) to assess the added value of fitting and predicting a model with a spatial effect. We assessed goodness‐of‐fit for each model by estimating Area Under the Curve (AUC), True Skill Statistic values (TSS), sensitivity and specificity for both models with and without a spatial effect included. Threshold values were determined by maximizing sensitivity and specificity for the model, and all metrics were calculated using the *presenceabsence* package in R (Freeman & Moisen, 2008). Finally, to better understand spatial differences in relative probability of presence across the region, we categorized relative probability of presence into quintile groups (i.e., 0–0.2 and 0.21–0.4) and estimated the proportion of the landscape in each ecoregion (per Albert, 1995) comprised of each category of occurrence.

## 3 RESULTS

### 3.1 Marten

**FIGURE 2:**
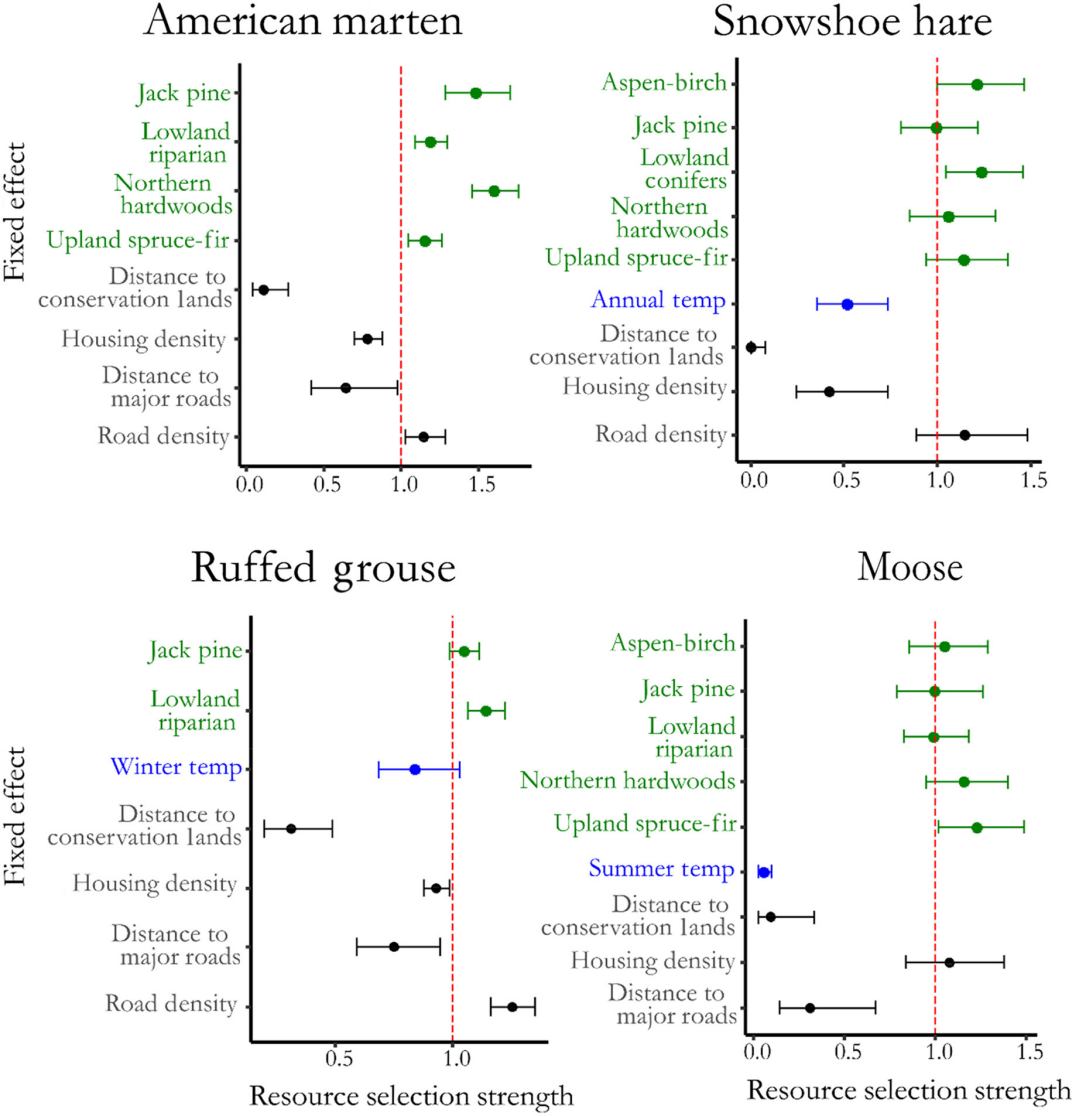
Forest plots indicating the relative selection strength of predictors in the top models used for predicting the probability of presence and range distribution of American marten (*Martes americana)*, snowshoe hare (*Lepus americanus*), Ruffed Grouse (*Bonasa umbellus*) and moose (*Alces alces*) in the northern lower peninsula and upper peninsula of Michigan. Dots indicate the mean of the posterior distribution and error bars indicate the extent of 95% credible intervals for the posterior distribution for each predictor. Credible intervals overlapping the red dotted line (1) indicate a nonsignificant relationship, while intervals to the left of the line indicate a negative relationship, and intervals to the right indicate positive relationships. Colors indicate the type of fixed effect (green—habitat, blue—climate, black—anthropogenic).

**FIGURE 3:**
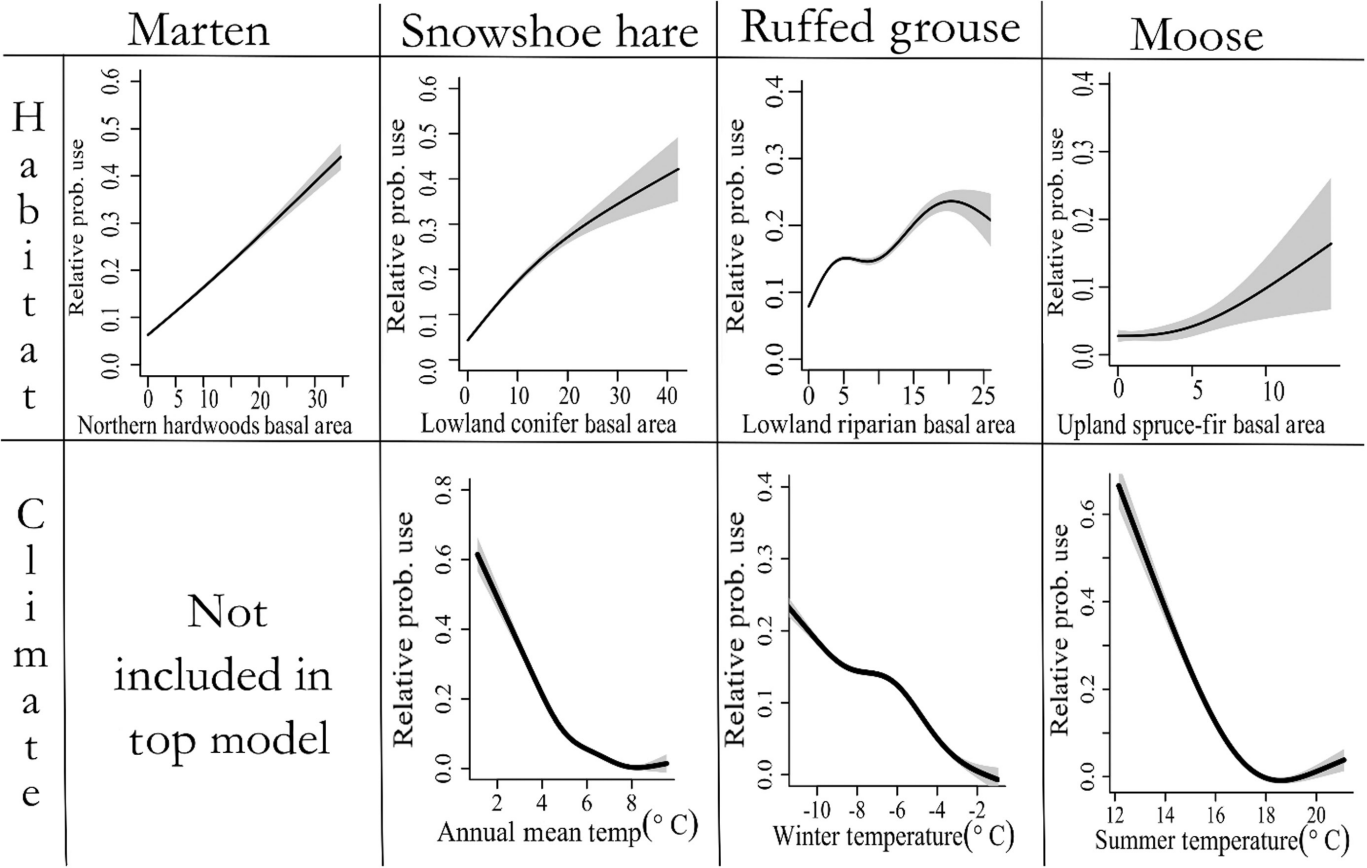
Effect plots for the habitat and climate variables with the largest effect size from habitat selection analysis of American marten (*Martes americana*), snowshoe hares (*Lepus americanus*), Ruffed Grouse (*Bonasa umbellus*), and moose (*Alces alces*) in northern Michigan. Curves are created by fitting a generalized additive model to the fitted values and predictor values for available points, and grey borders indicate 95% credible intervals. Basal area measurements are in m^2^/hectare.

**FIGURE 4:**
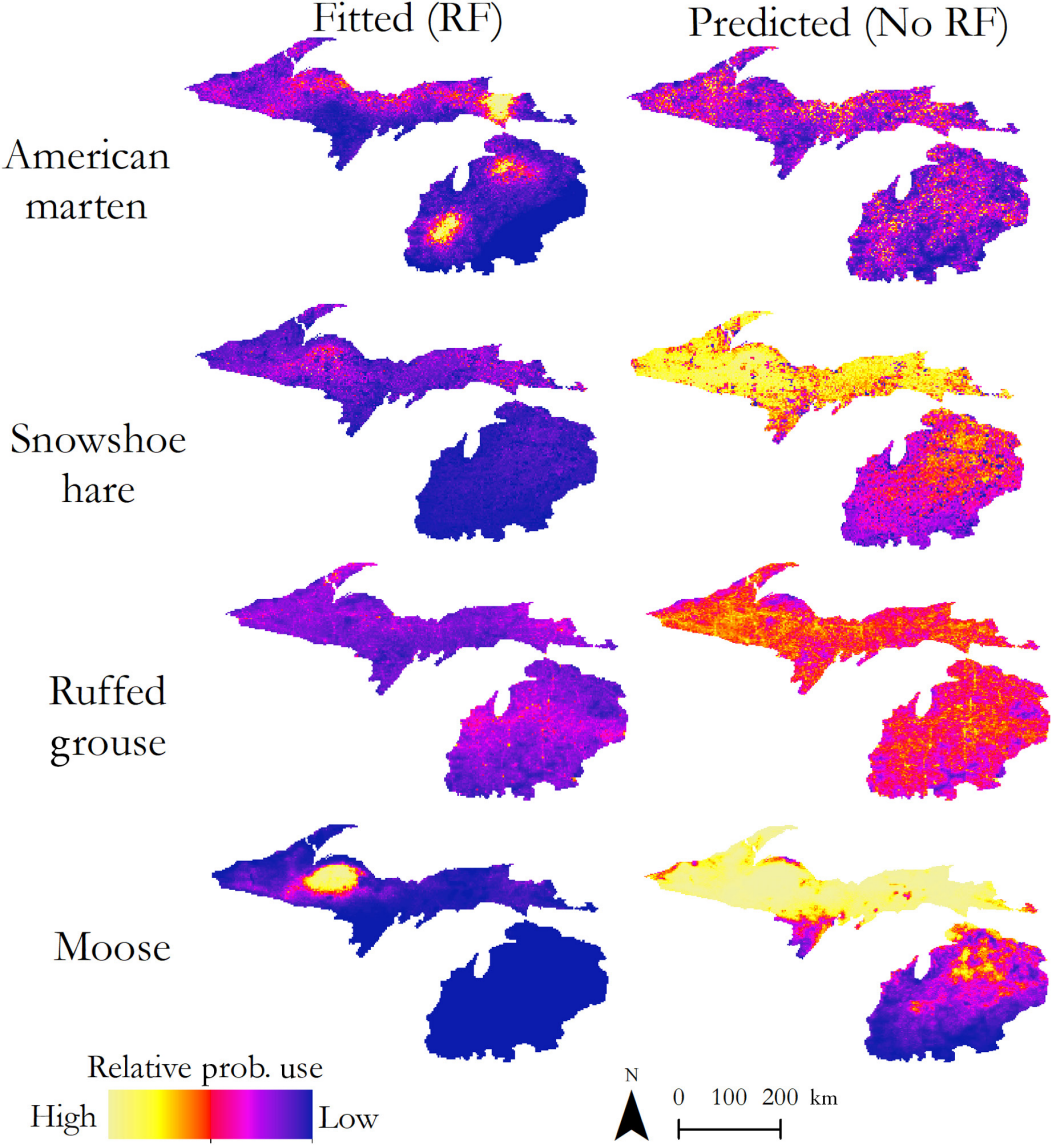
Predicted relative probabilities of use for American marten (*Martes americana*), snowshoe hares (*Lepus americanus*), Ruffed Grouse (*Bonasa umbellus*) and moose (*Alces alces*) in the northern lower peninsula and upper peninsula of Michigan from the fixed effects of the top model from habitat, climate and anthropogenic predictors, with the left‐hand model for each species including a random spatial effect (RF).

Marten showed positive associations with multiple forest cover types (Figure 2), as the relative probability of presence increased with increased basal area of Jack Pine–Red Pine (*β* = 0.39, 95% CI = 0.25, 0.53), Lowland Riparian (*β* = 0.18, 95% CI = 0.09, 0.26), Northern Hardwoods (*β* = 0.47, 95% CI = 0.37, 0.56), and Upland Spruce‐Fir (*β* = 0.14, 95% CI = 0.048, 0.24) forest types. Relative probability of presence increased 1.5× (95% CI: 1.3, 1.7) for each increase in basal area of 5.3 m^2^/ha of Jack Pine–Red Pine species, 1.19× (95% CI: 1.1, 1.3) for each increase in basal area of 4.43 m^2^/ha of Lowland Riparian, 1.6× (95% CI: 1.5, 1.8) for each increase in basal area of 6.86 m^2^/ha of Northern Hardwoods species, and 1.2× (95% CI: 1.0, 2.7; Figure 2) for each increase in basal area of 2.36 m^2^/ha of Upland Spruce‐Fir species. The variable with the largest effect size on marten probability of presence was the positive relationship between Northern Hardwood basal area (Figure 3). None of the models containing climate variables outperformed the top model with only habitat variables (Table 2). Probability of marten presence increased as road density increased (*β* = 0.14, 95% CI = 0.02, 0.25), and decreased as housing density (*β* = −0.25, 95% CI = ‐0.36, −0.13) and distance to conservation lands (*β* = −2.2, 95% CI = ‐3.1, −1.3) increased. Our spatial models indicated strong differences between the fitted (RF) and predicted (No RF) models (Figure 4). Both models showed broad extents of suitable habitat conditions throughout the Upper Peninsula, though the fitted model showed a stronger latitudinal gradient in relative presence than the predicted model (Figure 4). Examination of the random field shows several areas, particularly in the Lower Peninsula where spatial variation remained high after fitting the model, indicating unexplained variation due to an effect not included in the model fitting process (Figure S3). Examination of ecoregions indicates high amounts of suitable habitat and climatic conditions across the Upper Peninsula, but less located in the Lower Peninsula and Northern Green Bay Lobe (Figure S4). Model validation indicated an improved fit for the fitted model over the predicted model, with AUC values of 0.83 and 0.97 for the nonspatial model and spatial‐effect model, respectively (Table 3). Additionally, the fitted model had higher values for TSS, sensitivity, and specificity (Table 3).

**TABLE 2:** Model selection results for hierarchical modeling of presence only data of American marten (*Martes americana*), snowshoe hares (*Lepus americanus*), ruffed grouse (*Bonasa umbellus*) and moose (*Alces alces*) in the northern lower peninsula and upper peninsula of Michigan. Only those models within ≤2.0 ΔWAIC of the top model are shown.

	Model	WAIC	ΔWAIC
American Marten			
Habitat (H)	Jack Pine + Lowland Riparian + Northern Hardwoods + Upland Spruce‐Fir	4638.38	0.00
Climate (C)	H + NULL	4638.38	0.00
Anthropogenic	H + C + Distance to Major Rds + Housing Density + Rd Density + Distance to Cons Lands	4157.34	0.00
Snowshoe hare			
Habitat (H)	Aspen‐Birch + Jack Pine + Lowland Conifers + Northern Hardwoods + Upland Spruce‐Fir	877.60	0.00
Climate (C)	H + Annual Temp	859.80	0.00
	H + Winter Temp	859.92	0.12
Anthropogenic	H + C + Housing Density + Rd Density + Distance to Conservation Lands	835.87	0.00
	H + C + Housing Density + Distance to Conservation Lands	836.19	0.32
	H + C + Housing Density + Distance to Major Rds + Distance to Conservation Lands	836.69	0.82
	H + C + Housing Density + Rd Density + Distance to Major Rds + Distance to Cons Lands	837.42	1.55
Ruffed grouse			
Habitat (H)	Jack Pine + Lowland Riparian	6379.02	0.00
	Lowland Riparian + Upland Spruce‐Fir	6379.23	0.21
	Aspen Birch + Jack Pine + Lowland Conifers + Northern Hardwoods + Upland Spruce‐Fir	6380.34	1.32
	Lowland Riparian + Northern Hardwoods + Upland Spruce‐Fir	6380.38	1.36
	Aspen Birch + Lowland Riparian	6380.51	1.50
Climate (C)	H + Winter Temp	6364.18	0.00
Anthropogenic	H + C + DistMajRd + Housing Density + Road Density + Distance to Cons Lands	6287.80	0.00
Moose			
Habitat (H)	Aspen‐Birch + Jack Pine + Lowland Riparian + Northern Hardwoods + Upland Spruce‐Fir	9657.74	0.00
Climate (C)	H + Summer Temp	1023.37	0.00
Anthropogenic	H + C + Distance to Major Rds + Housing Density + Distance to Cons Lands	950.22	0.00
	H + C + Distance to Major Rds + Rd Density + Housing Density + Distance to Conservation Lands	951.78	0.99

**TABLE 3:** Results from model validation of habitat selection models for American marten (*Martes americana)*, snowshoe hares (*Lepus americanus*), Ruffed Grouse (*Bonasa umbellus*) and moose (*Alces alces*) in the northern lower peninsula and upper peninsula of Michigan.

Species	Threshold		AUC		TSS		Sensitivity		Specificity	
	Fitted	Predicted	Fitted	Predicted	Fitted	Predicted	Fitted	Predicted	Fitted	Predicted
American marten	0.33	0.60	0.97	0.84	0.81	0.51	0.87	0.79	0.94	0.71
Snowshoe hare	0.89	0.87	0.91	0.89	0.75	0.71	0.94	0.95	0.8	0.76
Ruffed Grouse	0.16	0.14	0.77	0.67	0.42	0.25	0.7	0.77	0.72	0.48
Moose	0.29	0.98	0.98	0.97	0.89	0.84	0.93	0.91	0.96	0.93

*Note*: Comparisons are made between models including a random spatial effect (RF) and an identical model lacking the spatial effect (Non‐RF). Threshold values were calculated to maximize sensitivity and specificity values. Comparisons of goodness‐of‐fit were carried out using Area under the curve (AUC), True Skill Statistic (TSS), sensitivity and specificity.

### 3.2 Snowshoe hare

Snowshoe hare presence was positively associated with the increased basal area of Aspen‐Birch (*β* = 0.19, 95% CI = 0.0030, 0.38), and Lowland Conifer habitats (*β* = 0.21, 95% CI = 0.044, 0.38; Figure 3). The top‐ranked model (Table 2) also included variables for Jack Pine (*β* = −0.0077, 95% CI = −0.22, 0.20), Northern Hardwoods (*β* = 0.057, 95% CI = −0.16, 0.27), and Upland Spruce‐Fir (*β* = 0.13, 95% CI = −0.059, 0.32), but *β* coefficients had 95% credible intervals overlapping zero. An increase in basal area of 5.30 m^2^/ha of Aspen‐Birch and 4.96 m^2^/ha of Lowland Conifer species, respectively, increased relative probability of presence by 1.2× (95% CI = 1.0, 1.5; Figure 2). Hare presence was negatively affected by increasing annual mean temperature (*β* = −0.66, 95% CI = −1.02, −0.31; Table 2), with a 1.1°C increase in annual mean temperature resulting in a predicted decline in relative likelihood of use by half (RSS = 0.51, 95% CI = 0.36, 0.74). Hare presence was negatively associated with increasing housing density (*β* = −0.85, 95% CI = −1.40, −0.31) and distance to conservation lands (*β* = −5.7, 95% CI = −8.8, −2.5). The final top‐ranked model also included Road Density; however, the *β* coefficient's 95% credible interval also overlapped zero (*β* = 0.14, 95% CI = −0.12, 0.40) and we deemed it uninformative (Arnold, 2010). Fitted and predicted spatial models showed strong similarities and both predictions showed a mid‐to‐high probability of presence throughout the Upper Peninsula, with lower habitat suitability across the Lower Peninsula (Figure 4). Postmodel fitting, the random field showed only slight variation across the study region indicating that our model predictors explained much of the spatial variation present in the region (Figure S3). Examination of ecoregions indicated that probability of presence was high across the Upper Peninsula, but lower in the Lower Peninsula (Figure S4). Model validation indicated only minor improvements in model performance due to addition of the spatial effect, with only negligible improvements in AUC, TSS, sensitivity, and specificity values (Table 3).

### 3.3 Moose

Relative habitat suitability for moose was positively related to basal area of Upland Spruce‐Fir (*β* = 0.21, 95% CI = 0.015, 0.40; Figure 3). The top‐ranked habitat model also contained variables for Aspen‐Birch (*β* = 0.049, 95% CI = −0.16, 0.25), Jack Pine (*β* = −0.0034, 95% CI = −0.24, 0.23), Lowland Riparian (*β* = −0.0092, 95% CI = −0.19, 0.17), and Northern Hardwood (*β* = 0.014, 95% CI = −0.050, 0.34) forest cover types, but all had 95% credible intervals overlapping zero (Figure 3). An increase in basal area of 2.36 m^2^/ha of Upland Spruce‐Fir habitat increased relative probability of presence by 1.2x (95% CI = 1.0, 1.5; Figure 3). Moose presence steeply declined as mean summer temperatures increased (*β* = −3.0, 95% CI = −3.6, −2.3; Figure 3), and a 0.98°C increase in mean summer temperature resulted in a 95% decrease in relative probability of presence (Figures 2 and 3). Relative likelihood of use declined as distances to conservation areas (*β* = −2.4, 95% CI = −3.6, −1.1) and major roads increased (*β* = −1.2, 95% CI = −2.0, −0.40). Housing density was also included in the top‐ranked final model (Table 2), although the 95% credible interval overlapped zero (*β* = 0.073, 95% CI = −0.18, −0.32). Spatial predictions showed differences between fitted (RF) and predicted (No RF) models (Figure 4), with the predicted model indicating a large amount of suitable habitat across the Upper Peninsula, but most of the location data occurring primarily in the Northern Upper Peninsula (Figure 4). Similarly, the random field showed a high amount of spatial structure unexplained by the model variables, particularly in the Northern Upper Peninsula ecoregion (Figure S3), indicating the presence of other factors driving spatial structure in the Northwoods region. Examination of ecoregions, indicated high relative habitat suitability across the Upper Peninsula, excepting the Southwest Lake Superior Clay Plain and Northern Green Bay Lobe (Figure S4). Model validation indicated only small differences between the performances of models with and without spatial effects, with AUC, TSS, sensitivity, and specificity values being negligibly improved by inclusion of the spatial effect variable (Table 3).

### 3.4 Ruffed grouse

Ruffed grouse exhibited a positive relationship between habitat suitability and basal area of Lowland Riparian cover types (*β* = 0.13, 95% CI = 0.064, 0.20; Figure 3). An increase of 4.43 m^2^/ha in the basal area of Lowland Riparian species resulted in a 1.1× (95% CI = 1.1, 1.2; Figure 2) increase relative habitat suitability. The variable for Jack Pine was also included in the top‐ranked habitat model (Table 2), but had a 95% credible interval overlapping zero (*β* = 0.047, 95% CI = −0.01, 0.11). The top‐ranked climatic predictor was annual winter temperature; however, the 95% credible interval overlapped zero (*β* = −0.18, 95% CI = −0.38, 0.03) indicating it was only weakly informative. The top‐ranked model including anthropogenic effects indicated that probability of grouse presence increased as road density increased (*β* = 0.22, 95% CI = 0.15, 0.30) and decreased with increases in distance to major roads (*β* = −0.30, 95% CI = −0.53, −0.056), housing density (*β* = −0.07, 95% CI = −0.13, −0.015), and distance to conservation lands (*β* = −1.2, 95% CI = −1.6, −0.72). Both spatial predictions with and without the spatial effect indicated grouse distribution across the Upper and Lower Peninsulas, but that probability of presence was closely linked to linear features, namely roads (Figure 4). Additionally, the fitted model indicated a band of high relative probability of presence across the Lower Peninsula, while this band was absent in the predicted model (Figure 4). Despite these differences in visualization of predictive models, the random field showed a relatively homogenous surface indicating that the variables included in the model were likely sufficient for explaining present spatial structure (Figure S3). Examination of ecoregions indicated few areas with a high probability of presence (Figure S4), though the Southern Superior Uplands and Northern Highlands still maintained relatively high levels of probability of presence. We observed notable differences in model goodness‐of‐fit between our predictive models with and without the random spatial effect. Inclusion of the random field improved the model fit metrics AUC, TSS and specificity, though there was a slight decline in specificity (Table 3). Despite this improvement, both models had a relatively poor goodness‐of‐fit as compared to the other modelled species (Table 3).

## 4 DISCUSSION

As predicted, distributions of four climate sensitive wildlife species in the Northwoods region were driven by the effects of climate, habitat, and human activity. While the relative importance of climatic variables varied among species, the effect of habitat availability (portrayed as amounts of climate sensitive forest cover types) on occupancy probability affected all species. Indeed, the final model for each species contained multiple habitat predictors, and similarities among species were identified. Grouse and marten presence were positively associated with lowland riparian cover types, and moose and marten positively associated with upland spruce‐fir cover type. Additionally, snowshoe hares were positively associated with lowland conifer cover type, which was strongly correlated with lowland riparian cover type because Northern white‐cedar occurred in both. Finally, while jack pine–red pine appeared in the final models for all species, coefficient posterior means overlapped zero indicating that it was only weakly informative. However, preservation and conservation of jack pine–red pine stands benefit wildlife species, particularly if applied at optimal locations. For example, American marten in the northern Lower Peninsula select for den sites in large DBH trees located in mature red pine plantations, which is unique to this population (Sanders et al., 2017). As Jack Pine–Red Pine forests are vulnerable to the effects of climate change (Table 1), loss of these habitats will likely result in demographic consequences for this isolated population. Additionally, areas of high basal area likely represent older forests, while some species may preferentially use young forests (Pietz & Tester, 1983), which may not be apparent in our results. Important wildlife cover types in this study represent a range of vulnerabilities to climate change. Upland spruce‐fir is considered highly vulnerable to climate change due to sensitivity to temperature and precipitation changes, while lowland riparian is considered moderately vulnerable (Handler et al., 2014; Reich et al., 2022). While specific mechanisms threatening each of these cover types differ, generally each is threatened by changes to the hydrological cycle, insect or disease outbreaks and increased herbivory by white‐tailed deer (Handler et al., 2014). Due to the multitude of threats to their distinct cover types, it is likely that each of these vertebrate species will experience negative indirect effects from habitat loss due to climate change.

Contrary to predictions, temperature variables outperformed the winter habitat indices in all models where a climate variable was selected. Moreover, despite literature suggesting each of these species having some aspect of their demographics affected by snow or temperature conditions (Evans & Mortelliti, 2022; Shipley et al., 2020; Weiskopf et al., 2019; Zimova et al., 2016), only two of four species showed a strong relationship with climate variables, which conflicts with current knowledge of the ecology and natural history of these species. For instance, snowshoe hare are experiencing range contractions due to camouflage mismatch resulting from attenuated snow conditions (Mills et al., 2013; Wilson et al., 2019; Zimova et al., 2016), and we would expect snow to be the dominant driver of distributions. However, other studies have also determined temperature to be the top predictor of occupancy and survival (Burt et al., 2017), and observed contrasting seasonal effects of warming temperatures on hare densities, with warming winters increasing hare density and warming summers decreasing hare density (Kumar et al., 2022). The predictors for snow season length and annual mean temperature were highly correlated within our dataset, and we suspect that correlation is higher when aggregating annual values to a mean rather than looking at individual years. Additionally, there are multiple parameterizations used to define snow season length that vary in the definitions of onset and termination of snow season. While we used first and last occurrence of snow to determine season length based on the winter habitat indices (Gudex‐Cross et al., 2021), other studies have used seven or 14 consecutive days of snow presence or absence to define onset and termination respectively (Wilson et al., 2019; Zimova et al., 2016) or total days of winter snow cover (Kumar et al., 2022). While alternative parameterizations may have changed our results slightly, each of these parameterizations is capturing the same aspect of winter conditions and are likely highly correlated particularly when aggregated across multiple years as we have done. Similarly, marten have previously been associated with deep snow conditions (Evans & Mortelliti, 2022), yet we observed no relationship between marten habitat suitability and any climatic variables, including those describing snow.

While we had predicted that more mechanistic measurements of local climate, such as winter habitat indices, would outperform broad temperature metrics, there are several factors potentially affecting this result. First, at the spatial extent and resolution at which we examined these relationships all climate predictors were strongly correlated and were not included in the same candidate model. It is likely had we looked at finer resolutions (i.e., closer to the 500‐m resolution which the habitat indices were created at), and across a broader geographic range we may have seen more variability in the winter habitat indices and potentially stronger relationships. Furthermore, while these indices have been shown to be strong predictors of species richness (Gudex‐Cross et al., 2022), they may be less suited for constructing distribution models from presence only data. Although our analysis was unable to determine the more specific, mechanistic drivers of range contraction, we do show that in some circumstances simpler metrics such as temperature may serve as a valuable proxy for these mechanistic indices. This can be valuable as models predicting temperature under future climate change scenarios have higher confidence than those predicting precipitation (Kapnick & Delworth, 2013). Finally, recent advances in the modelling of climate‐sensitive species have shown that spatial non‐stationarity may be particularly important for populations along range boundaries and that the effect of climate variables on habitat suitability may vary spatially (Sultaire et al., 2022). Indeed, studies of snowshoe hares incorporating nonstationarity have indicated that at broad scales, snow cover defines distributions, but temperature modulates the strength of the relationship across space (Sultaire et al., 2022). Incorporating nonstationarity into species distribution models may result in more precise estimates of the effects of climatic variables, and improve our understanding of the interactions between space, climate, and habitat that define species' range boundaries (Humphreys et al., 2022). Nonstationarity is likely to be particularly important along range boundaries, as dynamics can often differ greatly from those in the core of distributional ranges (Sexton et al., 2009).

We observed strong anthropogenic effects on predicted occurrence of all focal species. Many of these relationships, such as the negative relationships with housing density observed by martens, hares and grouse, likely reflect the tendency of wildlife to avoid areas dominated by humans (Lewis et al., 2021); however, some effects may reflect bias in the sampling method in which location data were collected, a well‐known problem with unstructured citizen science data (Dickinson et al., 2010). Many wildlife species show spatial and temporal avoidance of areas of high human activity, typified by high housing and road density and accompanying anthropogenic noise and light pollution (Gaynor et al., 2018; Wilson et al., 2021). Martens, hares, and grouse exhibited evidence of this avoidance, with increased probability of presence near conservation areas and in areas of low housing density. These results match the ecology of these species, particularly in relation to habitat selection, with martens reliant on large tracts of forest with vertical structure, hares reliant on forest patches with high stem densities or complex understories(particularly young aspen and late‐seral conifers), and grouse reliant on early successional forests during spring and fall. These results also underscore importance of connectivity among conservation areas, as each of these species are expected to or are currently undergoing range contractions (Burt et al., 2017; Pomara & Zuckerberg, 2017). Increased connectivity may allow for prolonged persistence of populations along southern range edges by allowing for immigration and emigration among isolated patches within climate or habitat refugia and populations within the core of the species' range. We observed other relationships that more likely resulted from how occurrence data were assembled. For instance, the increased probability of grouse presence with increased road density likely stems from use of eBird citizen science data as the only data source for grouse presence. Similar relationships were observed for marten and moose, likely due to the proximity of trapping locations to accessible roads and the use of roadkill data for marten and moose, respectively. Indeed, moose typically exhibit scale and context‐dependent relationships with roads, showing avoidance of landscapes with high road density and avoiding major roads at finer scales, but potentially showing preference for smaller roads and trails when they facilitate movements in deep snow or allow access to forage and sodium (Beyer et al., 2013; Laurian et al., 2008; Wattles et al., 2018). Spatial bias is a well‐known problem within citizen science data collection (Tiago et al., 2017), and inclusion of additional data sources where surveys were conducted away from roads may have improved this model. It is also important to note that we only examined the presence of these species rather than population densities, and effects of climate and habitat on abundance rather than mere occurrence may reveal otherwise hidden effects on population dynamics. For instance, further examination of grouse abundance and population cycles are likely to reveal declines across much of the southern range boundary as declines have been observed in other regions and linked to climate variability (Pomara & Zuckerberg, 2017).

Our comparison between spatial models with and without use of a random spatial effect revealed some advantages of using random fields in fitting species distributions. Use of the spatial‐effect nominally improved model goodness‐of‐fit for most species, but was particularly important for American marten. This was likely due to the abundance of telemetry locations at a number of focal areas used in the marten dataset, which made accounting for spatial autocorrelation critical. Examination of the random field post‐model fitting revealed spatial regions where our predictors did not adequately predict the spatial structure observed from the data and provided further insight into each species' history in the study region that would have been unavailable otherwise. For example, fitted distributions of marten showed two discontinuous distributional patches in the Lower Peninsula, while these patches were absent in the predicted model containing only fixed effects. The martens detected in these patches are the result of translocations performed in the 1980s (Williams et al., 2007), with many of the occurrence records coming from follow‐up telemetry studies used for monitoring. These occurrence records represent the southernmost populations within the study area, and are potentially in suboptimal habitat and climatic space. Similarly, moose only occur in the Upper Peninsula, yet our results indicate potential habitat in the uppermost Northern Lower Peninsula. This is due to suitable climatic and habitat conditions in this area, rather than actual detections of moose, but indicate that at present this region could sustain a population of moose. However, suitable habitat/climate space along this southern edge is unlikely to persist given future projections of climate change in the region. The measurement of the random field and resulting large values in this region likely indicate that climate and habitat alone cannot fully explain the presence or absence of these populations, and management action (i.e., translocations and regulatory protection) must be accounted for to fully understand the drivers of species distribution. As community science data continue to become more commonly used and the use of presence‐only models continues to proliferate, random spatial effects may be an effective diagnostic tool to identify areas where unexplained spatial variation occurs and to evaluate alternative drivers of spatial distribution.

Our results provide insights into future management actions to benefit climate‐sensitive wildlife species along the southern edge of their range. While these species are affected directly by climate as well as habitat, climate change is a problem that is not manageable on a local scale, while habitat manipulation and preservation can be practiced at multiple scales and can sometimes buffer the negative impacts of climate change (Wilson et al., 2019), making it an appealing option for the management of wildlife species. Northern white‐cedar basal area was an important component of the habitat model for three of four species, but cedar are vulnerable to browsing by ungulate species such as white‐tailed deer, which are expanding their northern distribution in response to attenuated winter conditions (Weiskopf et al., 2019). Our results suggest that management to promote cedar regeneration and retention, particularly in disturbed areas where herbivory may inhibit regeneration, can provide benefits to multiple wildlife species in addition to facilitating conservation of cedar itself. Likewise, upland spruce‐fir was an important habitat component for multiple species, but is highly temperature sensitive and vulnerable to the effects of climate change (Handler et al., 2014). While management of this forest type may not be feasible across the entire study area, focusing on conservation and protection within Upper Peninsula ecoregions—particularly the Northern Highlands, Southern Superior Uplands, Northern and Eastern Upper Peninsula—may have the most benefit for vulnerable species. Current distribution maps indicate that these ecoregions have relatively high probability of presence by all species examined, and may serve as a suitable climatic refugia for these species as climate change continues to shift suitable climatic conditions poleward (Sirén et al., 2021; Sirén & Morelli, 2020). Actions such as reducing browsing by decreasing white‐tailed deer abundance (Villemaire‐Côté et al., 2022), and identifying potential areas for restoration where the effects of climate change are expected to be relatively minimized due to geography or other factors (Zenner & Almendinger, 2012) may slow the effects of climate change in this region on species composition.

Despite the labelling of each of our focal species as climate‐sensitive, contemporary responses to climate and habitat varied greatly between species indicating that future responses to climate change may differ as well. Evidence of these differential responses may already be observable when considering the variation of suitable habitat and climatic space along the southern edge of these species' ranges. Indeed, differences in the precise mechanisms that negatively influence fitness and survival can influence the velocity at which the distributional ranges of these species shift northward. Additionally, while we have looked at each of these species individually, climate change is also altering biotic interactions within these ecological communities (Blois et al., 2013), which will undoubtedly affect the population dynamics of these species as habitat and climatic conditions change. Nonetheless, our analyses have emphasized the importance of climate‐sensitive forest cover types for climate sensitive vertebrate species, highlighting the likelihood of both indirect and direct effects of climate change on these species. While climate change and its direct effects may be most actionable on global or regional scales, habitat management can occur at local scales and the indirect costs incurred by the effects of climate change on habitat can potentially be mitigated by effective management, slowing the velocity of climate change and potentially creating refugia for species unable to persist given the negative effects of climate change.

## AUTHOR CONTRIBUTIONS

**Evan C. Wilson:** Conceptualization (equal); data curation (lead); formal analysis (lead); methodology (equal); writing – original draft (lead); writing – review and editing (equal). **Stella Cousins:** Conceptualization (equal); funding acquisition (equal); project administration (equal); writing – review and editing (equal). **Dwayne Etter:** Data curation (equal); writing – review and editing (equal). **John M. Humphreys:** Methodology (equal); writing – review and editing (equal). **Gary J. Roloff:** Data curation (equal); methodology (equal); writing – review and editing (equal). **Neil Carter:** Conceptualization (equal); funding acquisition (equal); project administration (equal); writing – review and editing (equal).

## CONFLICT OF INTEREST STATEMENT

The authors declare no conflict of interest.

## Supporting information


Supplementary Figures:

## Data Availability

Data (Wilson et al., 2023a) are available in Dryad at https://doi.org/10.5601/dryad.08kprr55p. R code (Wilson et al., 2023b) is available on Zenodo at https://doi.org/10.5281/zenodo.7908523.
