# Supplementary material for: Habitat and climatic associations of climate‐sensitive species along a southern range boundary
Source: Ecol Evol. 2023 May 17;13(5):e10083. doi: 10.1002/ece3.10083 (PMC10191803; doi:10.1002/ece3.10083)
Supplement: Supplementary file 1 — Supplementary Figures: [file ECE3-13-e10083-s001.docx]

**Supplemental Information for:**

**Supplemental Figures:**

**
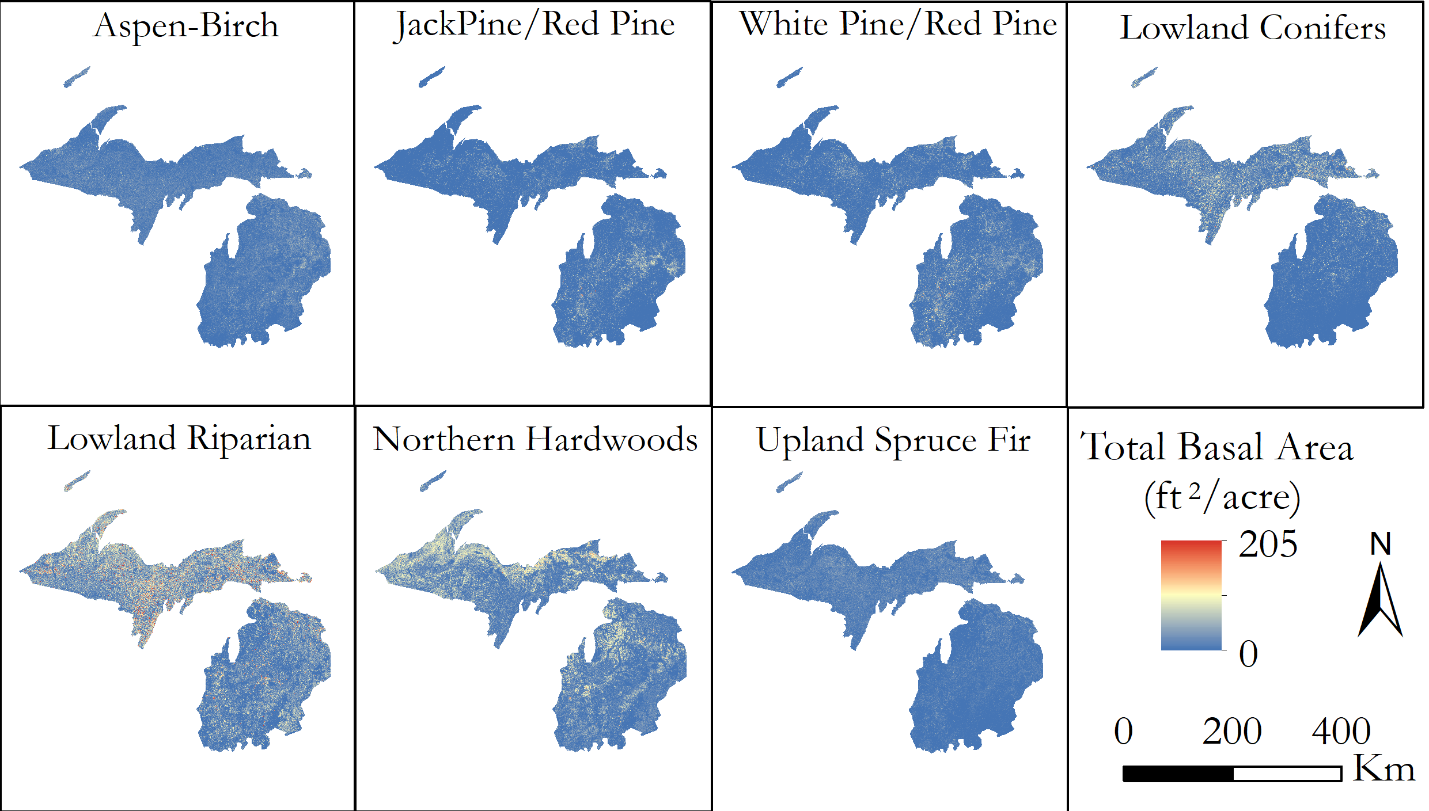
**

Fig S1: Distribution of habitat variables used in habitat selection analysis of moose, snowshoe hare, American marten and ruffed grouse.


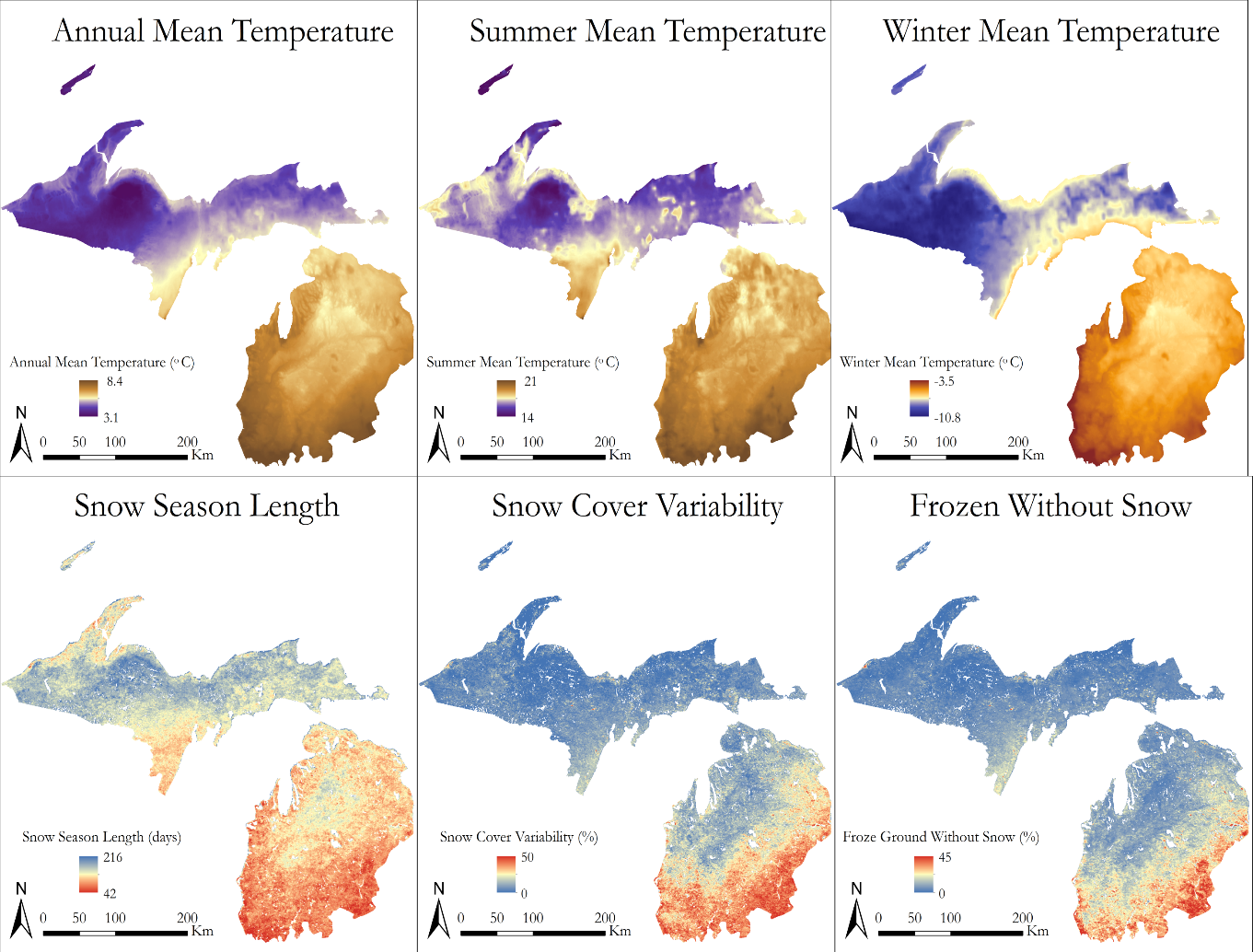


Fig S2: Distribution of climate variables used in habitat selection analysis of moose, snowshoe hare, American marten and ruffed grouse.


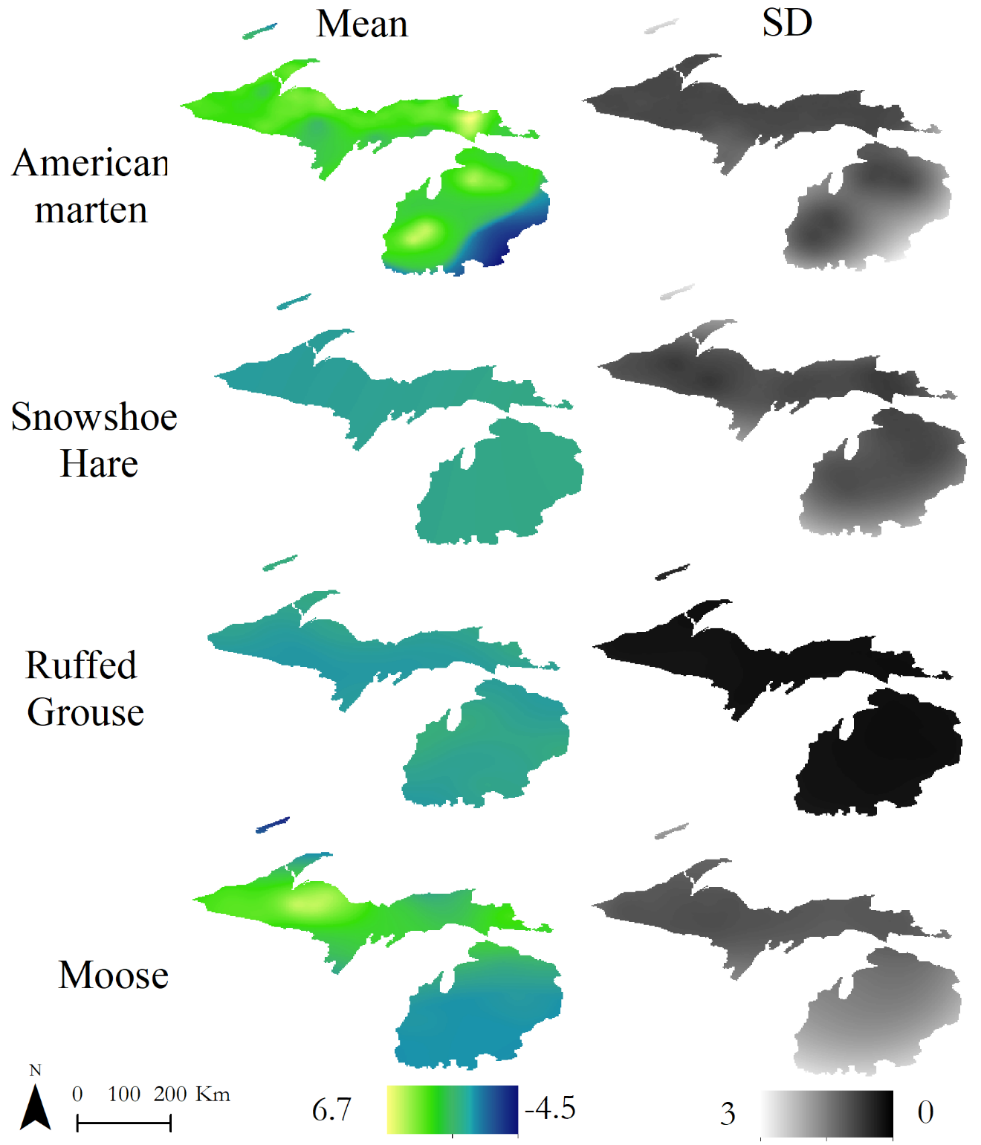


Figure S3: Post model-fitting visualization of the random field for distribution models of American marten (*Martes americana)*, snowshoe hares (*Lepus americanus*), Ruffed Grouse (*Bonasa umbellus*) and moose (*Alces alces*) in the upper Lower Peninsula and Upper Peninsula of Michigan. Values are unitless, but represent areas where predictors in the model explain less (high values) or more (low values) of the spatial variation present than expected.


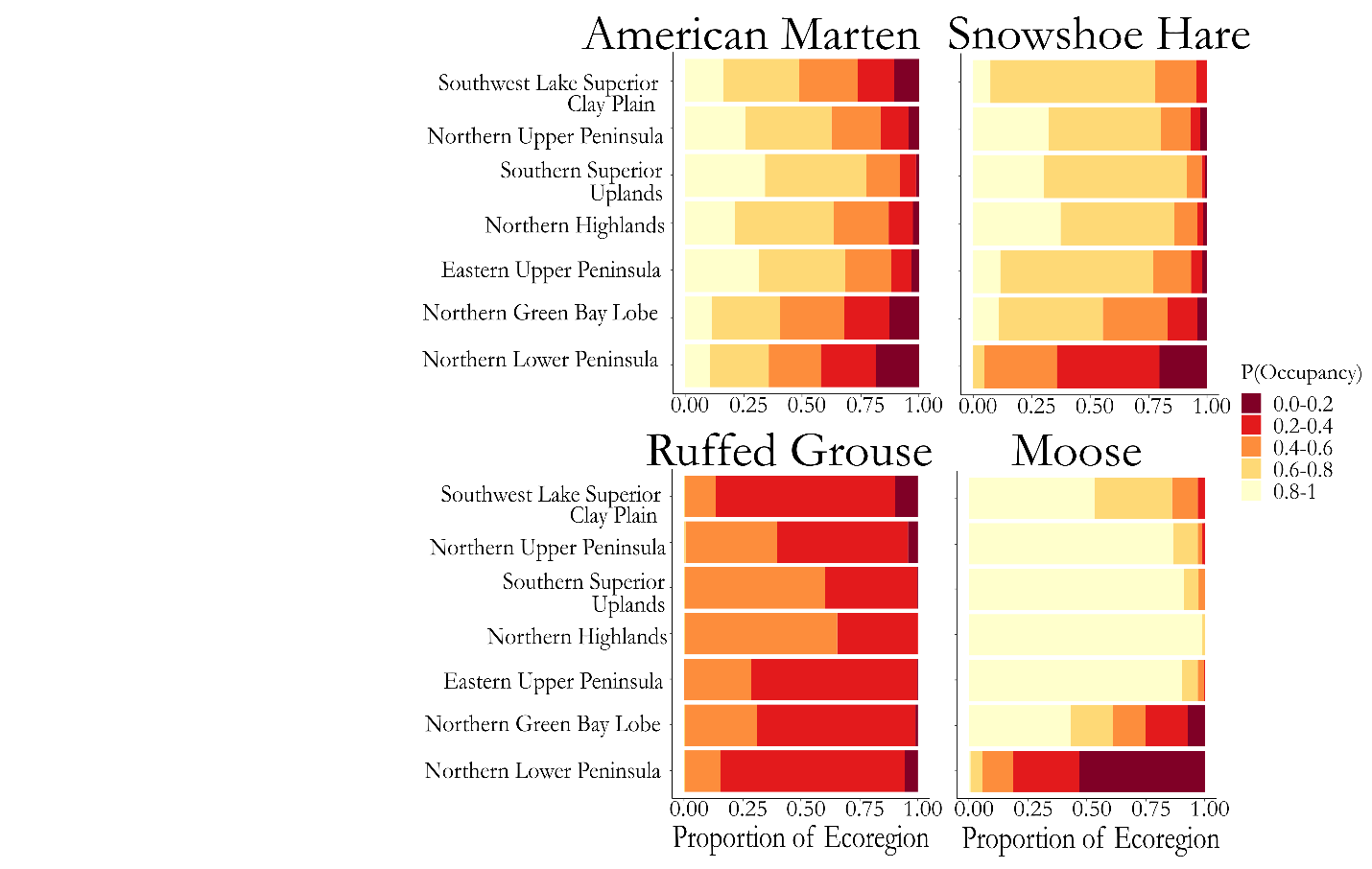


Figure S4: Stacked bar plots indicating the proportion of quintiles of relative habitat suitability from distributional predictions of occurrence for each ecoregion in the upper Lower Peninsula and Upper Peninsula of Michigan (per Albert 1995) for American marten (*Martes americana)*, snowshoe hares (*Lepus americanus*), Ruffed Grouse (*Bonasa umbellus*) and moose (*Alces alces*). Refer to Figure 1A for ecoregion map.

**Data Availability Statement:**

Data will be made publicly available in a Dryad repository, but is currently privately viewable for review at: https://datadryad.org/stash/share/0l5R3zsB2ZUcz9HtNAqVvb1I1nWMsW236BsvJHu6PPc
